# The Nutritional Properties, Chemical Compositions, and Functional Characteristics of the Aerial Parts of *Adonis coerulea*

**DOI:** 10.3389/fnut.2022.850714

**Published:** 2022-04-15

**Authors:** Lixia Dai, Bing Li, Xiaorong Yang, Yu Wang, Hu Pan, Jiyu Zhang, Xiaofei Shang

**Affiliations:** Key Laboratory of New Animal Drug Project, Key Laboratory of Veterinary Pharmaceutical Development of Ministry of Agriculture, Lanzhou Institute of Husbandry and Pharmaceutical Sciences, Chinese Academy of Agricultural Sciences, Lanzhou, China

**Keywords:** *Adonis coerulea* Maxim., nutritional ingredients, chemical compositions, PPAR-γ, antioxidant activity

## Abstract

The nutrition and active compounds from plants are very important to regulate the immunity of the body by improving the oxidant and inflammatory response. In this article, we aimed to investigate the nutritional profile and the phytochemical compositions of *Adonis coerulea*; the functional characteristics and its possible mechanism were studied. Results showed that the aerial parts of *Adonis coerulea* (ACAP) contained the abundant of proteins (16.15%) and the minerals (31.02.09 mg/100 g dried ACAP); promisingly, the content of essential amino acids (8.25%) and fatty acids (13,220.45 mg/100 g) also were obtained to regulate the immunity and prevent some chronic diseases. The methanol extract of ACAP played the anti-inflammatory activity *via* peroxisome proliferators-activated receptor (PPAR)-γ-mediated nuclear factor kappa B (NF-κB) pathway. Among the 18 identified compounds, linolenic acid from fatty acids and licochalcone A were active compounds by inhibiting nitric oxide (NO) production of RAW264.7 cells induced by lipopolysaccharide (LPS). The alleviation of inflammatory response results in the decrease of oxidative stress; ACAP showed the antioxidant activity by attenuating antioxidant enzymes, improving mitochondrial membrane potential and reactive oxygen species. These results highlight the potential of *A. coerulea* as a source of active ingredients in pharmaceutical industries.

## Introduction

The genus *Adonis* (Ranunculaceae) comprises 32 annual or perennial herbaceous species and distributes in temperate regions of the northern hemisphere of Europe and Asia (1). Some *Adonis* spp. were used as folk medicine in Europe and China to treat various diseases, such as cardiac enhancement function. Presently, many compounds were isolated from the plants of this genus, including cardiac glycosides, flavones, carotenoids, coumarins, and other structural classes, which presented the cardiac-enhancing, anti-inflammatory, antibacterial, antioxidant, antiangiogenic, diuretic, and acaricidal activities (2–5).

*Adonis coerulea* Maxim. is a perennial herbaceous species that grows in scrub and grassy slope areas and is distributed in northeast Tibet and the Sichuan, Qinghai, and Gansu provinces in China at altitudes of 2,300–5,000 m (6). In early 2011, we found that the aerial parts of this plant were used as folk medicine to treat skin disease in the Ruoergai region in our field investigation (7, 8). Due to the good safety *in vivo* and *in vitro* (8), the aerial parts of this species could be eaten by animals. However, the limited literature is available related to the enhancement of its food value, nutritional composition, and health benefits.

In this article, we objected to investigate the nutritional profile, chemical compositions, and the bioactivity of the aerial parts of *A. coerulea* (ACAP). First, the nutritional value, including proximate, mineral, amino acids, and fatty acids compositions, was evaluated. The chemical compositions was analyzed using ultra-performance liquid chromatography tandem mass spectrometry (UPLC-MS/MS); the anti-inflammatory and antioxidant activities and their possible mechanism were explored to advance its uses. This article may highlight its potential use and lay the foundation for the future development of aerial parts of *Adonis coerulea* (ACAP) as a sustainable natural resource.

## Materials and Methods

### Plant

*Adonis coerulea* Maxim. was collected from Tianzhu County in Gansu Province, China (37°11.4′ N latitude, 102°46.1′ E longitude, 3,200 m), in August 2019, and the raw material was identified by Prof. Zhigang Ma, Pharmacy College of Lanzhou University, China. A voucher specimen with accession number ZSY112-T1 was submitted to the Herbarium of Lanzhou Institute of Husbandry and Pharmaceutic Sciences, CAAS (Lanzhou, China).

### The Chemical Compositions of the Aerial Parts of *Adonis coerulea*

#### Proximate Composition

In this assay, the crude lipids, crude proteins, fiber, and ash of ACAP were determined. The crude lipids of the dried sample (2 g) were extracted using petroleum ether as a solvent and then measured using the Soxtec 2050 Automatic Soxhlet extraction analyzer (FOSS, Denmark) (9). Crude protein of the dried sample was determined according to the Kjeldahl method using the Kjeltec 8200 nitrogen analyzer (FOSS, Denmark) (AOAC, 10). The protein content was calculated by multiplying the nitrogen content by 6.25. Moisture and ash in ACAP also were determined according to the methods of GB.T 6435-2014/ISO 6496:1999 (11) and GB.T 6348-2007/ISO 5948:2002 (12), respectively. For moisture, after drying at 102°–105°C for 16–18 h to keep constant, the dry matter was weighed and moisture was calculated by using the formula (1); for ash, the sample was dried and heated at 525°–550°C, and then the ash was weighed. Three replicates were performed.


(1) $$\text{Moisture}={\text{W}}_{0}-{\text{W}}_{1}$$

where W_0_ represents the weight of dried sample, and W_1_ represents the weight of dry matter after drying at 102°–105°C for 16–18 h.

The digestible carbohydrate content was calculated by using the formula (2):


(2) Digestiblecarbohydrate=100-(%CL-%CP -%DF-%AS)

where CL represents the crude lipids, CP represents the crude proteins, DB represents the dietary fiber, and AS represents ash.

#### Mineral Composition

The dried sample (50 mg) was digested with HCl in a closed vessel microwave digestion system, and then the supernatant fluid was transferred to a volumetric flask for evaluating the mineral compositions of ACAP using an inductively coupled plasma mass spectrometry (ICP-MS) (Agilent 7900, United States). In this assay, calcium (Ca), phosphorus (P), copper (Cu), iron (Fe), selenium (Se), magnesium (Mg), manganese (Mn), potassium (K), sodium (Na), and zinc (Zn) were determined at 422.6, 213.6, 324.8, 248.3, 196.0, 285.2, 279.5, 766.5, 589.6, and 213.8 nm. Three replicates were performed, and the results for each mineral were presented as mg/100 g dried sample (9).

#### Amino Acid Composition

Before determining the amino acid composition, the dried sample (10 mg) was digested with 6 M HCl in a 20 ml of ampoule bottle, and then it was sealed and placed in an oven for hydrolysis at 110°C for 24 h. After filtering the solution, the supernatant was transferred in a bottle and placed in an evaporimeter to dry, and 5 ml of sodium citrate buffer (pH 2.2, *_*C*_*Na^+^ 0.2 mol/L) was added to dissolve it again to the concentrations of amino acid range of 50–250 nmol/L. Finally, the sample was centrifuged, and the supernatant was obtained. With respect to standard solution of amino acids (Sigma, United States), they were subjected to the Biochrom 30^+^ Automatic Amino Acid Analyzer (Biochrom, England) with an ion-exchange column for determining the contents of amino acids. The detector was set at 440 nm for proline and 570 nm for the other amino acids (13).

#### Fatty Acids Compositions

After mixing with the dried ACAP (20 mg), with internal standard (undecanoicacid, Sigma, United States, 2.0 mL) methanol solution and pyrogallol (100 mg) in a glass tube, the mixture was heated at 80°C for 1 h for saponification. Then, 15% boron trifluoride in methanol (7 ml) was used to converse the fatty acids to esters at 80°C for 1 h again, and hexane (15 mL) was added with saturated sodium chloride solution in this tube. After vortexing, the hexane layer was separated and transferred into a vial, and the derivatized fatty acids were determined by an autosampler gas chromatography (GC, 7890A, Agilent, United States) with a flame ionization detector.

Fatty acids were separated using an Agilent HP-88 column (60 m × 0.25 mm, 0.2 μm). Helium was used as the carrier gas, and the split ratio was 100:1. The oven temperature was held at 100°C for 13 min and raised to 180°C at a rate of 10°C/min and held for 6 min; subsequently, it was raised to 200°C at a rate of 1°C/min and held for 20 min; and it was raised finally up to 230°C at a rate of 4°C/min and held for 10.5 min. Fatty acids were calculated using fatty acid conversion factors from its methyl ester [AOAC Official Method 996.06 (modified), 14].

#### Ultra-Performance Liquid Chromatography Tandem Mass Spectrometry Analysis

To prepare the methanol extract, the ACAP (10 g) was soaked in methanol (100 ml) and then decocted at 80°C for 2 h three times. After combining, filtering, and concentrating, the methanol extract was dried in shadow and was used in the following assay. Three replicates were performed.

Subsequently, the methanol extract was dissolved in methanol and filtered through a 0.22 μm filter. The solution was obtained and subjected to an Agilent Technologies apparatus (1290 Infinity II, Agilent, United States) containing a quadrupole time-of-flight mass analyzer (Agilent 6530 QTOF, Agilent, United States) equipped with a dual electrospray ionization source (ESI) operating in negative and positive ion modes. An Aglient SB C_18_ RRHD column (2.1 × 150 mm, 1.8 μm) was applied in UPLC for separation the sample. The raw spectra was analyzed using the Mass Hunter Qualitative Analysis software. The solvent system was composed of 0.1% formic acid solution (A) and acetonitrile (B), and a gradient elution method was applied as follows: 0–45 min 2–100% B, 45%; 45–50 min 100% with a flow rate of 0.3 ml/min. The temperature of column was set at 40°C. The scan time was set at 2 spectra/s; the desolvation gas rate was set to 10 L/min at 350°C, and the nebulizer pressure was 45 psi; the fragment voltage and the capillary voltage were set at 135 V and 3.5 KV, respectively, and collision energy (CE) was 20 eV. The mass range of MS/MS was set at 2–1,000 m/z.

### Cytotoxicity

The primary mouse macrophage RAW 264.7 cells were cultured in medium prepared with 10% fetal bovine serum (FBS) and 90% Dulbecco’s modified eagle medium (DMEM) under a humidified incubator of 5% CO_2_ at 37°C. The cytotoxicity of the methanol extract of ACAP against RAW 264.7 cells was evaluated using the ZETA cell counting kit (ZETA Life, United States). After incubating RAW 264.7 cells (5 × 10^4^ cells/well) in 96-well plates for 24 h, ACAP (10–1,000 μg/ml, 10 μl) was added and incubated again for 24 h. Then, the cell counting kit-8 (CCK-8, 10 μl) was added and co-cultured for 30 min, and the absorbance was measured at 450 nm. Dimethyl sulfoxide (DMSO) (0.1%) was used as a control.

### Anti-inflammatory Assay

The anti-inflammatory activity was evaluated by determining the inhibitory effect of ACAP on the production of inflammatory cytokines in RAW 264.7 cells, which was induced by lipopolysaccharide (LPS). 500 μl of RAW 264.7 cells (5 × 10^4^ cells/well) were incubated in 48-well plates for 24 h, and then 500 μl of ACAP (10–100 μg/ml) and dexamethasone (DEX, positive agent) (10 μg/ml) were added and cultured for 1 h again, respectively. After adding LPS (2 μg/ml) for 24 h, the cells were collected to determine the levels of nitric oxide (NO), interleukin-1β (IL-1β), and tumor necrosis factor-*a* (TNF-*a*) using enzyme-linked immunosorbent assay (ELISA) kits from Nanjingjiancheng Bio (NJJCBIO, China) (15).

### Network Pharmacology

First, 20 compounds identified from ACAP were selected for predicting the possible drug targets using Swisstargetprediction (16), which were filtered by probability ≥ 0.12 and checked using the Uniprot database defined as Homo sapiens. Meanwhile, the “anti-inflammatory” was used as terms to search the related genes from the Genecards database (17), and the intersection between the anti-inflammatory-related targets and the predicted targets of ACAP was picked as common targets.

Subsequently, these common targets were submitted to the STRING 11.0 database (18) to construct a protein-protein interaction (PPI) network with a minimum required interaction score more than 0.4 confidence; the hub targets and core compounds were performed by constructing the “Compounds-Targets” network. Finally, the hub targets were imported into the Enrichr database (19) to perform functional annotation and enrichment analysis. Gene Ontology (GO) terms and KEGG pathways with *p* < 0.05 were conducted.

### Molecular Docking

Molecular docking between identified targets and active compounds of ACAP was performed using the DOCK 6.9 software in the Yinfo Cloud Platform. The grid score (kcal/mol) was calculated based on the best pose.

### Anti-inflammatory Activity of Compounds Identified From Aerial Parts of *Adonis coerulea*

The anti-inflammatory activities of compounds were evaluated by determining the inhibitory effect of ACAP on NO in RAW 264.7 cells induced by LPS. Cells (5 × 10^4^ cells/well, 500 μl) were incubated in 48-well plates for 24 h, and then 500 μl of linolenic acid, licochalcone A, vitexin, isoorientin, orientin, caprylic acid, L-phenylalanine (50 μg/ml), and DEX (5 μg/ml) were added and cultured for 1 h again, respectively. After adding LPS (5 μg/ml) for 24 h, the cells were collected to measure the level of NO using ELISA kits (NJJCBIO, China) (15).

### Peroxisome Proliferators-Activated Receptor Assay

Cells (5 × 10^4^ cells/well, 500 μl) were incubated in 48-well plates for 24 h, and then 500 μl of ACAP (10–100 μg/ml) and DEX (positive agent) (10 μg/ml) were added and cultured for 1 h again, respectively. After adding LPS (2 μg/ml) for 24 h, the cells were collected to determine the levels of peroxisome proliferators-activated receptor (PPAR-γ) using mouse peroxisome proliferators-activated receptor ELISA) kits from Nanjingjiancheng Bio (NJJCBIO, China).

### Western Blot Analysis

After treating with ACAP for 1 h, LPS (2 μg/ml) was added to stimulate RAW 264.7 cells for 24 h. The protein of cells was extracted and then separated on SDS-PAGE and transferred to polyvinylidene fluoride (PVDF) membranes, which were blocked in 5% bovine serum albumin for 1 h at room temperature and incubated overnight. Antinuclear factor-κB (IκB) alpha, anti-IκB alpha (phospho S36), anti-NF-κB p65, and anti-NF-κB p65 (phospho S536) were purchased from Abcam (Shanghai, China) for the immunoblotting, and the relative protein expression was quantified compared with the β-actin level.

### Antioxidant Activity

#### Cellular Antioxidant Activity

After incubating for 24 h, the supernatants of RAW 264.7 cells (2 × 10^5^ cells/well) were discarded, and 2 ml of ACAP was acceded and cultured for 1 h. Then, H_2_O_2_ (0.6 mM) was used to stimulate oxidant stress for 24 h; the cells were collected to determine the activities of superoxide dismutase (SOD) and catalase (CAT), and the content of malondialdehyde (MDA) using the relative assay kits (Solarbio, China). Vitamin C (Vc, 10 μg/ml) was used as the positive control.

### Measurement of Mitochondrial Membrane Potential

After 24 h of treatment with ACAP, H_2_O_2_ (0.6 mM) was used to stimulate RAW 264.7 cells for 24 h; the cell culture was replaced with JC-1 staining solution. After incubating at 37°C for 30 min in darkness and washed with PBS, the fluorescence was photographed using an LSM-800 with Airyscan using the JC-1 assay kit (Solarbio, China). For red fluorescent of J-aggregates and the fluoresce green of J-monomers, the wavelength of excitation/emission was set as 525/590 nm and 490/530 nm, respectively (20).

### Measurement of the Cellular Reactive Oxygen Species Production

After 24 h of treatment with ACAP, H_2_O_2_ (0.6 mM) was used to stimulate RAW 264.7 cells for 24 h, and then cells were incubated with 2 mM 2′,7′-dichlorodihydrofluorescein diacetate (DCFH-DA) (NJJCBIO, China) at 37°C for 30 min in darkness and photographed (excitation/emission 500/525 nm) using an LSM-800 with Airyscan. The fluorescence index was calculated for measuring the ROS production.

### Statistical Analysis

Results are expressed as mean ± standard deviation. Data were analyzed by one-way ANOVA, followed by the Dunnett’s test when the data involved three or more groups using GraphPad Prism 8.0.2. Differences were considered statistically significant at *p* < 0.05.

## Results and Discussion

### The Chemical Compositions of the Aerial Parts of *Adonis coerulea*

#### Proximate Compositions

As shown in Table 1, the main composition was carbohydrates (59.25 ± 5.79%), followed by proteins (16.15 ± 1.12%) and fiber (12.13 ± 0.99%). This result showed that the content of crude proteins was 16.15 g/100 g dried sample; ACAP provides the abundant of crude proteins for animals. Meanwhile, due to the high content of fiber, ACAP could be used as feed additives of animals (i.e., cattle) to advance the digestion. The content of lipid was lowest with 1.50 ± 0.34%. The relationship between the proximate compositions and harvest seasons should be investigated further.

**TABLE 1:** The proximate compositions, minerals compositions, amino acids and fatty acids of the aerial parts of *Adonis coerulea*.

Proximate compositions (%)		Minerals element (mg/100 g)		Amino acids (%)		Fatty acids (mg/100 g)	
Lipids	1.50 ± 0.34	Calcium	1680.00 ± 65.00	Gly	0.52 ± 0.17	C6	62.13 ± 7.85
Proteins	16.15 ± 1.12	Sodium	195.48 ± 10.12	Ala	1.18 ± 0.78	C12	20.89 ± 2.45
Moisture	10.11 ± 1.35	Potassium	871.28 ± 18.76	Val	0.98 ± 0.65	C13	54.62 ± 3.89
Fiber	12.13 ± 0.99	Iron	2.55 ± 0.45	Leu	0.84 ± 0.09	C14	60.14 ± 4.65
Ash	11.00 ± 0.87	Phosphorus	300.00 ± 12.59	Ile	0.84 ± 0.10	C14:1	78.53 ± 9.01
Carbohydrates	59.25 ± 5.79	Copper	0.59 ± 0.05	Pro	0.75 ± 0.12	C15	23.32 ± 3.93
		Manganese	6.97 ± 0.78	Ser	0.86 ± 0.23	C15:1	48.50 ± 5.56
		Magnesium	44.03 ± 3.78	Cys	–	C16	2591.53 ± 100.46
		Selenium	0.05 ± 0.00	Met	1.11 ± 0.21	C16:1	22.74 ± 1.12
		Zinc	1.14 ± 0.09	Thr	0.84 ± 0.32	C17	53.89 ± 7.54
		Total	3102.09 ± 88.67	Phe	1.30 ± 0.12	C18	164.02 ± 12.34
				Tyr	1.33 ± 0.23	C18:1	1063.51 ± 55.64
				Asp	0.97 ± 0.09	C18:2	3555.89 ± 43.25
				Glu	0.93 ± 0.04	C18:3	4539.29 ± 78.45
				Lys	1.14 ± 0.51	C20	13.20 ± 1.24
				Arg	1.28 ± 0.41	C20:1	17.30 ± 0.98
				His	1.20 ± 0.33	C20:2	22.97 ± 3.54
				Total	16.07 ± 4.21	C20:3	129.83 ± 9.93
				E-total	8.25 ± 2.36	C21	317.28 ± 21.72
						C22:6	30.21 ± 1.42
						C23	54.13 ± 7.25
						C24	141.64 ± 10.46
						C24:1	135.86 ± 12.78
						Total	13220.45 ± 140.23
						SFA	3556.79 ± 84.34
						MUFA	1366.46 ± 42.12
						PUFA	8279.20 ± 56.78

*SFA, saturated fatty acids; MUFA, monounsaturated fatty acids; PUFA, polyunsaturated fatty acids.*

*E-total, The total contents of eight essential proteins and one protein (His) for children.*

#### Mineral Compositions

In Table 1, we could see that ACAP is rich in mineral compositions, and the total contents was 3,102.09 mg/100 g of dried sample. The main mineral was Ca, and the content was 1,680.00 mg/100 g, next were K (871.28 mg/100 g), P (300.00 mg/100 g), and Na (195.48 mg/100 g). Ca, K, P, and other minerals were thought as essential ligands of some antioxidant enzymes and played the important role on the antioxidant function of body (21). Hence, ACAP could be used to protect the body from diseases. Meanwhile, Mg, Mn, Zn, Cu, Fe, and Se also were detected. This result indicated that ACAP could be thought as a feed additive because of the high content of Ca, K, P, and Mg and also provide the various minerals.

#### Amino Acid Compositions

Above study showed that ACAP contains the abundant of amino acids, which are important for the formation of protein and are necessary to keep balance the diet and provide the nutrient substances needed by the body (22). The result showed that sixteen of amino acids were detected, and the content of the total amino acids was 16.07%. Surprisingly, the essential amino acids in ACAP were up to 50% of the total amino acids with the content of 8.25%, which could not be produced and synthesized by the body and only gotten from food. Eight essential amino acids were detected, and the contents of Phe, His, Lys, and Met were more than 1%. ACAP contained the most abundant of hydrophobic amino acids (3.07%), which have many bioactive functions to protect the body by enhancing the immune function (13, 23). In addition, the valuable sulfur-containing amino acids (Met, 1.11%) also were found, which presented the antioxidant potential and might provide good effects against oxidative damage (24). This results indicated that ACAP was a good source of essential amino acids and could be used as food supplementary.

#### Fatty Acids Compositions

Essential fatty acids are necessary to the human diet and food (13, 22). They also are considered an important source of biological components for treating some inflammatory diseases and cancer, and compounds with polyunsaturated fatty acids (PUFAs) were proved to have the pharmacological potential (25). In this test, the fatty acids compositions were determined, and chromatograms are presented in Supplementary Figure 1.

As shown in Table 1, the total of fatty acids of ACAP was 13,220.45 mg/100 g and was higher than many plants used as food supplementary (13, 14, 26). The mono-unsaturated fatty acids (MUFA) and poly-unsaturated fatty acids (PUFA) could decrease the risks of human coronary heart disease (27). In this article, we found that the contents were up to 1,366.46 mg/100 g for MUFA and 8,279.20 mg/100 g for PUFA, respectively, and the most abundant of fatty acids were C18:3 and C18:2 with the contents of 4,539.29 mg/100 g and 355.89 mg/100 g, respectively. Considering that people could not synthesize omega-3 and omega-6 fatty acids, which were only absorbed and obtained from the diet (27, 28), ACAP could be used as good source of unsaturated fatty acids and would be beneficial to the human health to prevent some chronic diseases. Linoleic acid and α-linolenic acid, as well as a precursor of arachidonic acid, eicosapentaenoic acid, and docosahexaenoic acid, presented the cardiovascular protective effect, anti-inflammatory, and antioxidant activities (29). These fatty acids could bind to PPAR-γ, with PUFAs having higher binding affinity than saturated or monounsaturated fatty acids, which also could decrease TNF-α production. Hence, this anti-inflammatory effect of fatty acids may be related to the regulation of PPAR-γ and NF-kB pathways (25).

The Western diet commonly existed the high omega-6/omega-3 ratio (10–20:1) with the high content of omega-6 and low level of omega-3 fatty acids; this diet would cause the progression of some chronic autoimmune and degenerative diseases (30). Hence, the low ratios of 1:1–4:1 were recommended and would be beneficial to human health for medicinal uses (30). In our work, the low ratio of omega-6/omega-3 and palmitic acid:oleic acid were found with the values of 1.31:1 and 1:2.44; this result indicated that ACAP provides the health food ingredients and could reduce the risk of diabetes (31). In addition, we also found that ACAP be rich in C16 fatty acids with the content of 2,591.53 mg/100 g. This result indicated that SRP contained the essential fatty acids and nutritional quality and could be used to prevent some chronic cardiovascular diseases.

### Ultra-Performance Liquid Chromatography Tandem Mass Spectrometry Analysis

**FIGURE 1:**
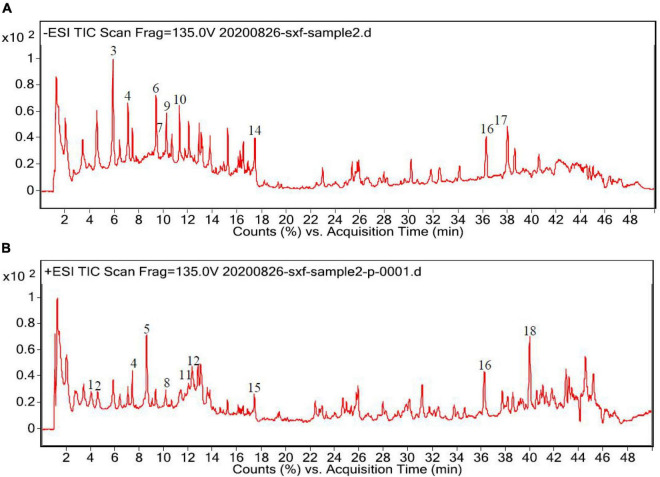
High-performance liquid chromatography (HPLC) chromatograms of the aerial parts of *Adonis coerulea* **(A)** for negative mode and **(B)** for positive mode. 1. L-phenylalanine, 2. caprylic acid, 3. licochalcone A, 4. vitexin, 5. magnoflorine, 6. isoorientin, 7. orientin, 8. 5′-hydroxypuerarin, 9. luteolin 5-glucuronide, 10. norwogonin-7-O-glucuronide, 11. bryoamaride, 12. syringaldehyde, 13. 4-methylumbelliferyl glucuronide, 14. 3-Rha-7-Rha quercetin, 15. strophanthidin, 16. linolenic acid, 17. linoelaidic acid, 18. acacetin-7-O-rutinoside.

Subsequently, the chemical compositions of ACAP were analyzed using UPLC- MS/MS, and the LC/MS chromatographic peak of both negative and positive ions were presented in Figure 1. The data on the retention time, molecular ion, main MS^2^ fragments, tentative chemical compounds, and molecular formula of ACAP were described in Table 2.

**TABLE 2:** Characterization of chemical constituents of the aerial parts of *Adonis coerulea* identified by ultra-performance liquid chromatography tandem mass spectrometry (UPLC-MS/MS).

No.	Rt	Mode	Experimental mass	Ion fragment (MSMS)	Compounds	Formula	Mass
1	4.06	[M-H]^+^	166.0862	120.0807	L-Phenylalanine	C_9_H_11_NO_2_	165
2	4.59	[M-H]^+^	145.1224	109.1014 83.0860 55.0552	Caprylic acid	C_8_H_16_O_2_	144
3	6.40	[M-H]-	337.1489	337.1517 157.0885 119.0359 59.0157	Licochalcone A	C_21_H_22_O_4_	338
4	7.42	[M-H]^+^	433.1340	127.0390 103.0392	Vitexin	C_21_H_20_O_10_	432
	7.47	[M-H]-	431.1175	431.1005 341.0690 311.0587 283.0639			
5	8.59	[M-H]^+^	342.1704	342.1704 297.1126 237.0908 58.0661	Magnoforine	C_20_H_23_NO_4_	341
6	9.31	[M-H]-	449.1082	449.1082 413.0870 383.0761 329.0657 299.0550	Isoorientin	C_21_H_20_O_11_	450
7	9.41	[M-H]-	447.0919	447.0968 357.0643 327.0587 283.0639	Orientin	C_21_H_20_O_11_	448
8	10.52	[M-H]^+^	433.1128	433.1128 397.0914 337.0698 313.0704 283.0601	5‘-Hydroxypuerarin	C_21_H_20_O_10_	432
9	10.66	[M-H]-	461.0714	461.0744 285.0423	Luteolin 5-glucuronide	C_21_H_18_O_12_	462
10	12.02	[M-H]-	445.0807	445.0807 269.0475 175.0261 113.0251	Norwogonin-7-Oglucuronide	C_21_H_18_O_11_	446
11	12.32	[M-H]^+^	679.5128	679.5128 661.5002	Bryoamaride	C_36_H_54_O_12_	678
12	12.80	[M-H]^+^	183.0780	98.9847 80.9744	Syringaldehyde	C9H10O4	182
13	12.83	[M-H]-	351.0714	163.0416 143.0363 119.0566	4-Methylumbelliferyl glucuronide	C_16_H_16_O_9_	352
14	17.19	[M-H]-	593.2956	44.9998	3-Rha-7-Rha Quercetin	C_27_H_30_O_15_	594
15	17.20	[M-H]^+^	405.2272	369.2085 323.2002 277.1967 239.1773 187.1488 152.0553 125.0600 95.0855	Strophanthidin	C_23_H_32_O_6_	406
16	36.20	[M-H]-	277.2190	277.2190	Linolenic acid	C_18_H_30_O_2_	278
	36.13	[M-H]^+^	279.2318	81.0705			
17	38.53	[M-H]-	279.2327	279.2327	Linoelaidic acid	C_18_H_32_O_2_	280
18	39.92	[M-H]^+^	593.1856	593.1856, 445, 283	Acacetin-7-O-rutinoside	C_28_H_32_O_14_	592

In Figure 1, we can see that about 30 compounds were observed in the total ion chromatogram. By comparing the mass spectrometric data of the literature and the natural product library, only eighteen compounds were identified, namely, flavonoids, amino acids, fatty acids, cardiac aglycone, alkaloids, terpenoids, phenols, and others. Other unknown compounds should be identified further to found the possible active ingredients. Among the identified compounds, most of the compounds belong to flavonoids, including licochalcone A, isoorientin, orientin, luteolin-5-glucuronide, 5′-hydroxypuerarin, vitexin, norwogonin-7-O-glucuronide, 3-rha-7-rha-quercetin, and acacetin-7-O-rutinoside (32); one amino acid, phenylalanine (33), three fatty acids, caprylic acid, linolenic acid, and linoelaidic acid (34, 35) were also detected from ACAP. In addition, one alkaloid magnoflorine (36), one terpenoid bryoamaride (37), one cardiac aglycone strophanthidin (38), and one coumarins 4-methylumbelliferyl glucuronide (39) were identified from this species. Isoorientin, orientin, and strophanthidin were also found by previous reports (38, 40). In addition, this result was consistent with the result of the total phenol and flavonoid analyses, and the content of flavonoids in ACAP was more than the content of phenols. Flavonoids, widely existed in plants, possessed the wide range of biological activities, such as antioxidant, anti-inflammatory, antibacterial, and immune regulation (41). Hence, we hypothesized that ACAP may have the possible anti-inflammatory and antioxidant activities.

### Cytotoxicity

**FIGURE 2:**
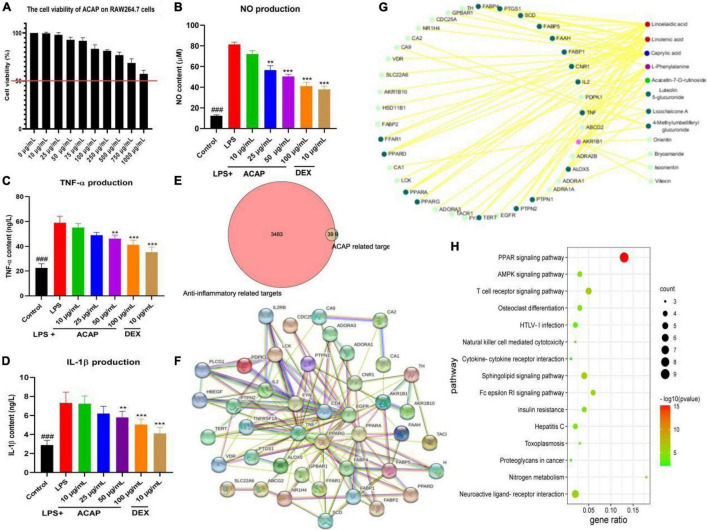
The cell viability of aerial parts of *A. coerulea* (ACAP) against RAW264.7 cells **(A)**, and the nitric oxide (NO) content **(B)**, tumor necrosis factor (TNF)-α content **(C)**, interleukin (IL)-1β content **(D)** in RAW264.7 cells induced by lipopolysaccharide (LPS); the common targets between ACAP related and anti-inflammatory targets **(E)**, the core hub of targets **(F)**, the target-compounds network **(G)**, and the KEGG pathway **(H)** in the network pharmacology assay (^###^represent the significant difference between control group and model group, *p* < 0.001; **p < 0.01 represent the significant difference between model group and drug treated group; ***p < 0.001).

The safety of ACAP was investigated by evaluating the cytotoxicity of ACAP against RAW 264.7 cells. Result showed that at the concentrations of 0–1,000 μg/ml, ACAP presented the safety *in vitro*. Even at 1,000 μg/l, the cell viability was more than 50% (Figure 2A).

### Anti-inflammatory Activity

Macrophages stimulated by LPS and other foreign substances would produce and release the proinflammatory cytokines, which engaged into the process of inflammatory damage (42). In this study, the anti-inflammatory activity of ACAP *in vitro* was investigated by determining the inhibitory effect on the production of inflammatory cytokines.

As a well-known inflammatory cytokines, NO is released by activated macrophages through the formation of peroxynitrite with superoxide anion and inflammatory response. Figure 2B showed that after the stimulation by LPS, the content of NO in RAW264.7 cells (81.40 μM) was rapidly increased compared with control group (12.41 μM, *p* < 0.001); however, the release of NO was significantly inhibited after the treatment of ACAP; the contents were 72.09 μM for 10 μg/ml, 56.59 μM for 25 μg/ml, 50.39 μM for 50 μg/ml, and 41.09 μM for 100 μg/ml (*p* < 0.001). In Figure 2C, we could see that after the treatment of ACAP, the levels of TNF-*a* in RAW 264.7 cells also were decreased compared with the model group in a dose-dependent manner, which plays an important role in inflammation formation. In addition, IL-1β mainly participated in the immune response and tissue repair in response to inflammatory diseases; the result showed that ACAP could decreased the inflammatory response induced by LPS by reducing the produce of IL-1β in a dose-dependent manner (Figure 2D). These results showed that ACAP presented the significant anti-inflammatory activity in a dose-dependent manner by decreasing the production of proinflammatory cytokines.

### Network Pharmacology

A network pharmacology was performed to explore the potential mechanism of action of ACAP. Eighteen compounds identified from ACAP was used as a candidate to collect the potential targets. In total, 48 targets acted with 12 compounds were screened out from the SWISSTARGET database, and 3,512 targets related anti-inflammatory were obtained; however, only 39 common targets were filtered out between inflammatory targets and compounds targets for the further study (Figure 2E).

Subsequently, a PPI network of the common targets was constructed using the String database, the total of 45 nodes and 152 edges were obtained, respectively, and the average degree of node was 6.76. The targets PPARA, EGFR, TNF, and PPARG with more edges and nodes were located in the core hub (Figure 2F). A compound-target network was established and constructed to obtain the potential mechanism, and 39 putative hub targets and 12 core compounds were identified. Among of compounds, linolenic acid and linoelaidic acid have the highest degree value (16), and the target AKR1B1 has the high degree of interaction with other targets and compounds (Figure 2G).

GO analysis showed that these genes were enriched in the regulation of inflammatory response, negative regulation of lipid localization, and negative regulation of lipid storage and other biological processes and participated in cellular components, such as membrane microdomains, membrane raft; these genes were involved in cytokine receptor binding, cytokine activity, carboxylic acid binding, protease binding, and others were involved in molecular functions. This result suggested that the targets of SRP against inflammatory related to regulation of the inflammatory response and cytokines. KEGG pathway analysis showed that the potential targets of active compounds in ACAP were mainly enriched in PPAR (peroxisome proliferators-activated receptors) signaling pathway, T-cell receptor signaling pathway, neuroactive ligand-receptor interaction, AMPK signaling pathway, and others (Figure 2H). These results indicated that SRP presented antiliver inflammatory activity by regulating the PPAR-mediated signaling pathways, and further studies were performed to prove this hypothesis.

### Active Compounds

**FIGURE 3:**
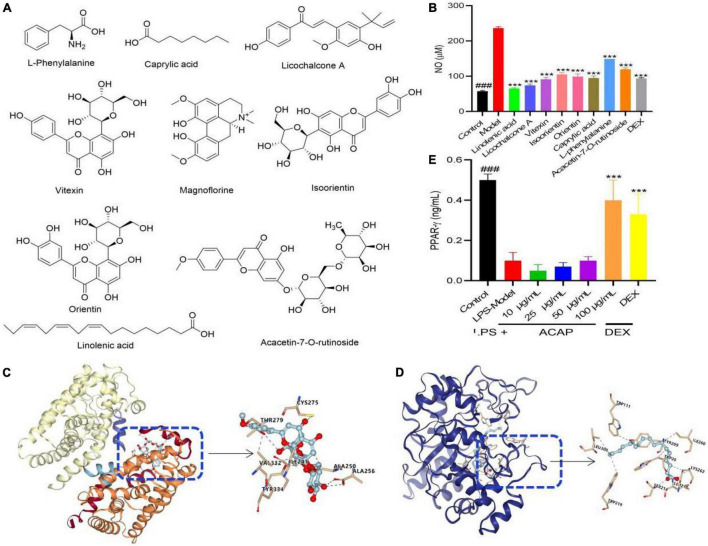
The potential chemical structure of active compounds identified from ACAP **(A)**, the inhibitory effect of eight compounds on NO production **(B)**, the affinity and binding sites between the target AKR1B1 and the compound linolenic acid **(C)**, the target AKR1B1 and the compound acacetin-7-O-rutinoside and AKT1 **(D)** in the molecular docking test, and the activation effect of ACAP on PPAR-γ **(E)**. ^###^Represent the significant difference between control group and model group, *p* < 0.001; ***p < 0.001 represent the significant difference between model group and drug treated group.

To verify the result of network pharmacology and find the active compounds of SRP, the anti-inflammatory activities were evaluated using the NO assay. As shown in Figure 3A, all compounds from ACAP significantly inhibited the NO production of RAW 264.7 cells induced by LPS (*p* < 0.001). Linolenic acid presented the best anti-inflammatory activity with the NO content of 65.34 μM, followed by licochalcone A (73.76 μM), vitexin (91.26 μM), and caprylic acid (95.01 μM). Orientin, isoorietin, and acacetin-7-O-rutinoside have the weak inhibitory effect on NO, whereas, NO contents of the control and DEX were 57.51 μM and 93.76 μM, respectively (Figures 3A,B). Considering that the oral bio-availability of only linolenic acid and licochalcone A were more than 40%, these results indicated that above compounds have the synergistic effect against anti-inflammatory activity for SRP, linolenic acid, and licochalcone A as active compounds.

### Molecular Docking

To prove the result of network pharmacology, the molecular docking between the targets AKR1B1 (PDB 5OUK) and PPAR (PDB 3VI8) and active compound linolenic acid and acacetin-7-O-rutinoside were conducted to prove their affinity and find the binding core, respectively. The result showed that linolenic acid bonded to with Leu (212, 300), Ser (214), Lys (262), Ile (260), and Trp (111, 219) of AKR1B1 with the high affinity, the grid score, and the internal energy of -75.79 kcal/mol (Figure 3C). Acacetin-7-O-rutinoside has the high affinity to the target PPAR with the grid score of -87.66 kcal/mol, it could bind to Tyr (334), Ala (256), Thr (279), and Cys (275) with hydrogen bond, to Val (332), Ile (241), and Ala (250) with hydrophobic interaction (Figure 3D). This result indicated that two active compounds played the potential anti-inflammatory activity *via* PPAR and AKR1B1, respectively.

### The Effect of Aerial Parts of *Adonis coerulea* on Peroxisome Proliferators-Activated Receptor

PPARs are ligand-activated transcription factors of nuclear hormone receptor superfamily, which play essential roles in the regulation of cellular differentiation, development, and metabolism and tumorigenesis. As one of the subtypes, PPAR-γ participated in the inflammatory response *via* the regulation of NF-κB. The activated PPAR-γ is translocated into the nucleus to bind with NF-κB, and then the NF-κB-PPAR-γ complex was formed, which cannot bind to the promotor region of DNA; thereby, gene expressions of proinflammatory mediators were suppressed (43). As shown in Figure 3E, ACAP could increase the level of PPAR-γ in RAW 264.7 cells induced by LPS in a concentration-dependent manner, especially for high concentration (100 μg/ml). This result proved the result of the network pharmacology assay. Considering that many fatty acids could bind to PPAR-γ, this result indicated that ACAP played the anti-inflammatory activity by activating PPAR-γ and then inhibiting the gene expressions of proinflammatory mediators, such as linolenic acid.

### Western Blot Analysis

**FIGURE 4:**
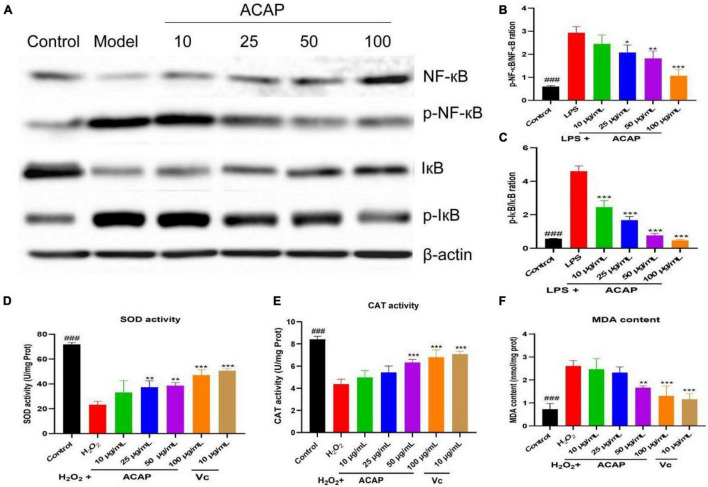
The western blot assay for determining the expression of IκB, NF-κB, and Akt **(A)**, the p-NF-κB/NF-κB ration **(B)**, the p-IκB/IκB ration **(C)**, and the SOD activity **(D)**, catalase (CAT) activity **(E)**, and MDA content **(F)** in RAW264.7 cells induced by LPS (^###^ represent the significant difference between control group and model group, *p* < 0.001; *p < 0.05 represent the significant difference between model group and drug-treated group; ** for *p* < 0.01; *** for *p* < 0.001).

The IkappaB kinase (IKK) complex was activated and led to the phosphorylation and degradation of the inhibitory IκB proteins and then stimulate activation of NF-κB to regulate the production of proinflammatory factors, including NO, TNF-α, IL-1β, and IL-6 (43). Hence, NF-κB axis is important to the expression of proinflammatory genes, and the modulation was thought as a good strategy to the treatment of inflammatory diseases by inhibiting proinflammatory gene expression. To prove the result of network pharmacology and explore the potential mode of action, the expressions of phosphorylation of IκB (p-IκB)/IκB and the phosphorylation of NF-κB (p-NF-κB)/NF-κB were investigated. As shown in Figures 4B,C, compared with the mode group, the rations of p-IκB/IκB and p-NF-κB/NF-κB of RAW 264.7 cells induced by LPS were decreased after the treatment of ACAP in a dose-dependent manner, especially for p-IκB/IκB (*p* < 0.001) (Figures 4A–C).

### Antioxidant Activity

Macrophages stimulated by H_2_O_2_ would cause the oxidant stress by elevating ROS, MDA, and attenuating antioxidant enzymes. SOD and CAT provide first-line cellular protection contributing to prevent cellular damage and maintain a balance between free radical production and oxidative stress after stimulated by oxidative stress (44). After the treatment of ACAP, the activity of SOD in RAW 264.7 cells was significantly activated compared with the model group in a dose-dependent manner (*p* < 0.01). At the concentrations of 100 μg/ml, the SOD activities were 47.12 U/mg Prot (*p* < 0.001), respectively, similar to the positive agent Vc of 50.68 U/mg Prot at 10 μg/ml (*p* < 0.001) (Figure 4D). At the high concentrations of 50 and 100 μg/ml, the CAT activity also was significant increased compared with the model group (*p* < 0.001) (Figure 4E). As well as the similar dose-dependent manner on CAT, ACAP decreased the elevated production of MDA in cells stimulated by H_2_O_2_ at the high concentrations (*p* < 0.001) (Figure 4F), which would be released when responding to oxidant stress. These results indicated that ACAP has the cellular antioxidant activity by activating the SOD and CAT activities and scavenging free radicals from macrophages induced by H_2_O_2_.

### Measurement of the Cellular Mitochondrial Membrane Potential

**FIGURE 5:**
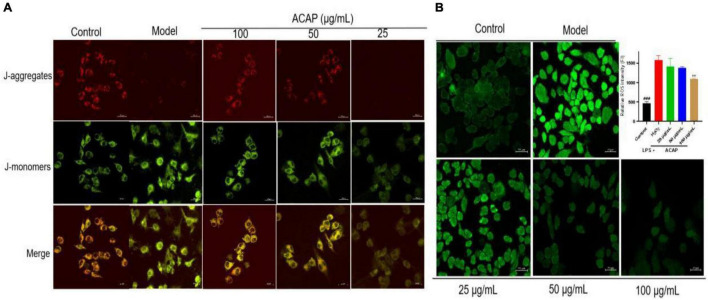
The fluorescence for measuring the MMP level **(A)** and the cellular ROS production **(B)** in RAW264.7 cells treated by ACAP (^###^represent the significant difference between control group and model group, *p* < 0.001; **p < 0.01 represent the significant difference between model group and drug-treated group).

In this test, the red fluorescence was seen as an aggregated pattern of energized mitochondria, and the green fluorescence was presented as a monomeric pattern for depolarized mitochondria. In Figure 5A, after the treatment of ACAP, the intensity of red and green fluorescence was reversed in a dose-dependent manner and showed higher red fluorescence and lower green fluorescence; however, cells induced by LPS presented the remarkably lower red fluorescence and higher green fluorescence. This result indicated that ACAP could improve the MMP depletion induced by LPS.

### Measurement of the Cellular Reactive Oxygen Species (ROS) Production

**FIGURE 6:**
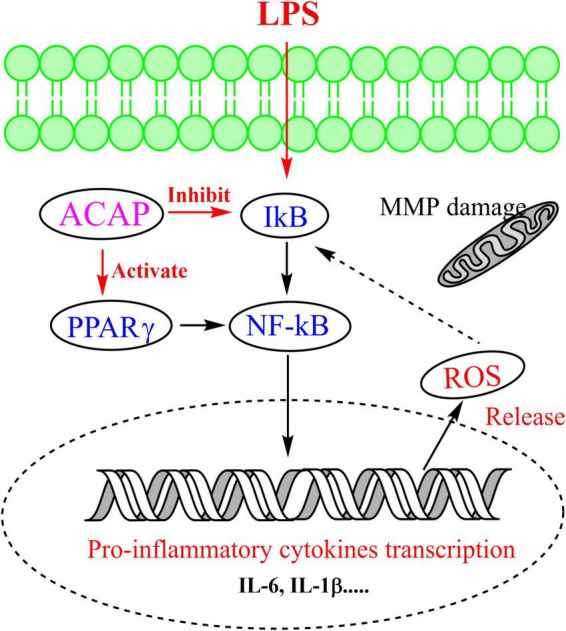
The possible anti-inflammatory mechanism of ACAP.

The oxidant response to exogenous substance would increase cellular ROS production (45). Hence, the cellular ROS production in RAW 264.7 cells was evaluated by determining the intensity of green fluorescence, which was stained by DCFH-DA. Compared with the control group (FI 464.05), the fluorescence intensity (FI) in the model group was significantly enhanced with the value of 1,583.13. However, after the treatment of ACAP, the green fluorescence become vague, and the intensities were decreased in a dose-dependent manner. The inhibition rates were 43.25% for 100 μg/ml (*p* < 0.01), 25.63% for 50 μg/ml, and 14.89% for 25 μg/ml. This result illustrated that ACAP inhibited the ROS production and accumulation of RAW264.7 cells induced by H_2_O_2_ (Figure 5B). Combined with the above result, we thought that ACAP played the anti-inflammatory and antioxidant activities by inhibiting PPAR-γ mediated NF-κB signaling pathway (Figure 6). The further studies should be carried out to prove this mode of action of ACAP and its active compounds.

## Conclusion

The ACAP was rich in the promising and valuable of nutrients and contained the abundant of flavonoids with potential anti-inflammatory and antioxidant activities *via* PPAR-γ-mediated NF-κB pathway. Linolenic acid from fatty acids and licochalcone A were active compounds by binding to PPAR-γ and then inhibiting the gene expressions of proinflammatory mediators. *A. coerulea* could be regarded as a source of nutritional ingredients and natural agent to regulate immunity for the pharmaceutical industries.

## Data Availability Statement

The original contributions presented in the study are included in the article/Supplementary Material, further inquiries can be directed to the corresponding author/s.

## Author Contributions

XS and LD: conceptualization. LD: methodology. XY: data curation. LD: writing–original draft preparation. BL: visualization. LD, YW, and HP: investigation. XS and JZ: supervision. HP: software and validation. XS and JZ: writing–reviewing and editing.

## Conflict of Interest

The authors declare that the research was conducted in the absence of any commercial or financial relationships that could be construed as a potential conflict of interest.

## Publisher’s Note

All claims expressed in this article are solely those of the authors and do not necessarily represent those of their affiliated organizations, or those of the publisher, the editors and the reviewers. Any product that may be evaluated in this article, or claim that may be made by its manufacturer, is not guaranteed or endorsed by the publisher.

## Supplementary Material

Supplementary Figure 1 The GC chromatograms of the aerial parts of Adonis coerulea.
