# Supplementary figures and images for: The Nutritional Properties, Chemical Compositions, and Functional Characteristics of the Aerial Parts of Adonis coerulea
Source: Front Nutr. 2022 Apr 15;9:850714. doi: 10.3389/fnut.2022.850714 (PMC9053748; doi:10.3389/fnut.2022.850714)

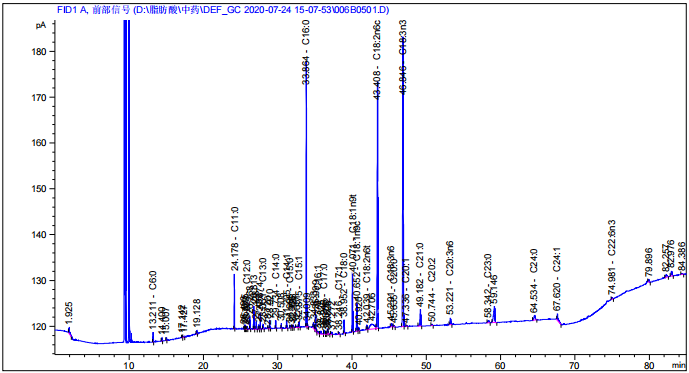

Supplement: Supplementary Figure 1 — The GC chromatograms of the aerial parts of Adonis coerulea. [file Image_1.PNG]
